# Study of Complete Genome Sequences of Rotavirus A Epidemics and Evolution in Japan in 2012–2014

**DOI:** 10.3389/fmicb.2019.00038

**Published:** 2019-01-31

**Authors:** Yoshiki Fujii, Yen Hai Doan, Yoshiyuki Suzuki, Toyoko Nakagomi, Osamu Nakagomi, Kazuhiko Katayama

**Affiliations:** ^1^Department of Virology II, National Institute of Infectious Diseases, Tokyo, Japan; ^2^Graduate School of Natural Sciences, Nagoya City University, Nagoya, Japan; ^3^Department of Hygiene and Molecular Epidemiology, Nagasaki University, Nagasaki, Japan; ^4^Laboratory of Viral Infection I, Kitasato University, Tokyo, Japan

**Keywords:** rotavirus, Japan, full-genome, next-generation sequencing, reassortment, Bayesian analysis

## Abstract

A comprehensive molecular epidemiological study using next-generation sequencing technology was conducted on 333 rotavirus A (RVA)-positive specimens collected from six sentinel hospitals across Japan over three consecutive seasons (2012–2014). The majority of the RVA isolates were grouped into five genotype constellations: Wa-like G1P[8], DS-1-like G1P[8], G2P[4], G3P[8] and G9P[8]. Phylogenetic analysis showed that the distribution of strains varied by geographical locations and epidemic seasons. The VP7 genes of different G types were estimated to evolve at 7.26 × 10^-4^–1.04 × 10^-3^ nucleotide substitutions per site per year. The Bayesian time-scaled tree of VP7 showed that the time to the most recent common ancestor of epidemic strains within a region was 1–3 years, whereas that of the epidemic strains across the country was 2–6 years. This study provided, for the first time, the timeframe during which an epidemic strain spread locally and within the country and baseline information needed to predict how rapidly RVAs spread.

## Introduction

Rotavirus A (RVA) is a major cause of gastroenteritis in infants and young children worldwide. In 2013, RVA caused 215,000 deaths in children under 5 years of age globally (Tate et al., 2016). RVA imposes a huge burden even on developed countries, including Japan (Sanchez-Fauquier et al., 2006; Hull et al., 2011; Banyai et al., 2012; McDermid et al., 2012; Lanata et al., 2013; Nakagomi et al., 2013; Collins et al., 2015).

The RVA genome contains 11 gene segments of double-stranded RNA that encode six structural (VPs) and six non-structural proteins (NSPs) (Estes and Greenberg, 2013). To classify the RVAs, a specific genotype is assigned to each of the 11 genome segments, according to predefined nucleotide sequence identity cutoff values (Matthijnssens et al., 2008a,b, 2011). The classification system denotes the VP7-VP4-VP6-VP1-VP2-VP3-NSP1-NSP2-NSP3-NSP4-NSP5/6 genes of an RVA strain as a descriptor Gx-P[x]-Ix-Rx-Cx-Mx-Ax-Nx-Tx-Ex-Hx (x indicates genotype number), respectively. Human RVAs have been mostly classified into three genogroups, based on the genotype constellations (Nakagomi et al., 1989). The Wa, DS-1 and AU-1 genogroups are described as G1-P[8]-I1-R1-C1-M1-A1-N1-T1-E1-H1, G2-P[4]-I2-R2-C2-M2-A2-N2-T2-E2-H2, and G3-P[9]-I3-R3-C3-M3-A3-N3-T3-E3-H3, respectively (Matthijnssens et al., 2008a,b, 2011). The G1, G3, G4, G9, and G12 viruses are usually grouped into Wa-like constellation, and G2 and G8 viruses are usually grouped into DS-1-like constellation (Heiman et al., 2008).

Recently, two live attenuated RVA vaccines were introduced in Japan (Rotarix^®^, GlaxoSmithKline Biologicals, Belgium in November 2011 and RotaTeq^®^, Merck & Co., Inc., United States in July 2012). Although RVA vaccines were not part of a routine immunization schedule in Japan, coverage was estimated to be at least 39% in 2012–2013 (Hashizume et al., 2015). The current RVA vaccines are effective (Lambert et al., 2009; Leshem et al., 2014b; Karafillakis et al., 2015), but the selective pressure of a vaccine may induce an epidemic strain shift. Therefore, we began a comprehensive molecular epidemiological study to monitor the RVA epidemics in Japan since 2012. In the first year of surveillance (2012), we reported the predominance of the unusual G1P[8] strain, which possesses a DS-1-like genotype constellation (DS-1-like G1P[8]: G1-P[8]-I2-R2-C2-M2-A2-N2-T2-E2-H2), in Japan (Fujii et al., 2014). Surprisingly, this strain with an unusual genotype constellation spread nationwide to become predominant (46.7%), and thus, this DS-1-like G1P[8] strain should be monitored continuously. A previous report (Fujii et al., 2014) focused on an emerging DS-1-like G1P[8] strain. In the present study, we performed comprehensive molecular epidemiological research, based on next-generation sequencing (NGS) of RVA strains in 2013–2014 and added published results from 2012, to generate a phylogenetic tree. Moreover, a Bayesian time scale analyses was conducted to better understand the epidemics in Japan and the evolutionary history of RVA.

## Materials and Methods

### Sample Collection

Surveillance was conducted at six sentinel hospitals in Hokkaido prefecture (NTT East Japan Sapporo Hospital and Otaru Kyokai Hospital), Akita Prefecture (Yuri Kumiai General Hospital), Aichi Prefecture (Konan Kosei Hospital), Kyoto Prefecture (Nantan General Hospital) and Yamaguchi Prefecture (Yamaguchi University Hospital) during three seasons (2012–2014) (Supplementary Figure S1). Three hospitals in Akita, Aichi and Kyoto participated throughout the entire period. The hospital in Yamaguchi Prefecture participated in 2013 season and into 2014. Two hospitals in Hokkaido Prefecture participated only in 2013. A total of 789 stool specimens were collected from children aged < 5 years who were hospitalized for acute gastroenteritis. Gastroenteritis was defined as three or more passages of watery diarrhea or looser-than-normal stool within 24 h. Specimens were collected after written informed consent was obtained from the patients or their guardians for the donation of samples. The typical RVA season in Japan is January to July. No RVA-positive stool was found from August to December in this study. In 2012, 237 specimens were collected from February to July as previously described (Fujii et al., 2014). In the 2013 season, 427 specimens were collected from January to July. In the last season (2014), surveillance began in January. Although the epidemic peak in Japan is March and April, sample collection was stopped by March 2014 because of the end of the fiscal year. Thus, only 125 specimens were collected from January to March in the 2014 season. Approximately 10% stool suspensions in PBS (10 mM, pH 7.2) were examined for RVA antigen with an ELISA kit, Rotaclone^®^ (Meridian Diagnostics, Cincinnati, OH, United States). ELISA-negative samples were excluded from analysis.

### cDNA Library and Nucleotide Sequencing

Viral RNA was extracted from stool suspensions with the Direct-zol RNA MiniPrep kit (Zymo Research, Irvine, CA, United States) according to manufacturer’s instructions as previously described (Fujii et al., 2014). NGS was performed as described (Dennis et al., 2014; Doan et al., 2016). Briefly, a 200-bp fragment library was constructed for each sample with the NEBNext Ultra RNA Library Prep Kit for Illumina v1.2 (New England Biolabs, Ipswich, MA, United States), according to manufacturer’s instructions. A 151-cycle paired-end-read sequencing run was conducted on a MiSeq desktop sequencer (Illumina, San Diego, CA, United States) using the MiSeq Reagent Kit v2 (300 cycles). Sequence data were analyzed using CLC Genomics Workbench Software v7.0.3 (CLC Bio, Aarhus, Denmark) and deposited in the DDBJ/GenBank databases (LC172271-LC174338). The NGS analysis yielded several sequences that lacked one or both ends of segments. This result usually depends on amount of virus in the stool specimen.

### Genotyping and Phylogenetic Analysis

Genotypes of the 11 genome segments were determined by the RotaC 2.0 automated genotyping tool for RVA (Maes et al., 2009). Near-full-length genome sequences were aligned with reference sequences using CLUSTAL W that was included in the MEGA software package, version 7.0.18 and the MAFFT multiple sequence alignment software program, version 7.0 (Katoh et al., 2009). Sequence alignments and phylogenetic analysis were performed using MEGA 7 software (Tamura et al., 2013). Genetic distances were calculated with the Tamura three-parameter at a nucleotide level, and phylogenetic trees were constructed by the Maximum Likelihood Method with 1000 bootstrap replicates. Reference sequences were retrieved from GenBank and the lineage designations defined based on previous studies for G1 (Arista et al., 2006; Le et al., 2010), G2 (Doan et al., 2015), G3 (Wang et al., 2014), and G9 trees (Phan et al., 2007).

### Bayesian Evolutionary Analysis by BEAST

Evolutionary rates and time of most recent common ancestors (tMRCA) were determined for the VP7 gene of RVAs by the Bayesian Markov chain Monte Carlo (MCMC) method implemented in BEAST v1.8.1 (Drummond et al., 2012). The model used for BEAST analysis was T93+G. A strict clock and coalescent exponential growth model (Drummond et al., 2002) were used. MCMC runs were carried out for 200 million generations to achieve convergence with sampling every 1000 steps. Convergence was assessed from effective sample size after a 10% burn-in using Tracer software v1.6.^1^ Only parameters with an effective sample size > 200 were accepted. Maximum clade credibility trees were annotated with Treeannotator and viewed with FigTree v1.4.2^2^.

### Ethics Statement

This study protocols were approved by the medical research ethics committee of the National Institute of Infectious Diseases for the use of human subjects (No. 335).

## Results

### Genotype Distribution Based on Full-Genome Sequencing

A total of 789 stool specimens were collected, and 333 RVA-positive samples were identified by ELISA screening: the detection rates were 45.6% (108/237) in 2012, 49.4% (211/427) in 2013, and 11.2% (14/125) in 2014. The genotype data of 108 samples in 2012 were previously reported (Fujii et al., 2014). In the present study, 225 RVA strains collected in 2013 and 2014 were analyzed by NGS, and the genotype constellations of all samples were determined. Most of the samples were grouped into five genotype constellations: G1-P[8]-I1-R1-C1-M1-A1-N1-T1-E1-H1 (Wa-like G1P[8]), G1-P[8]-I2-R2-C2-M2-A2-N2-T2-E2-H2 (DS-1-like G1P[8]), G2-P[4]-I2-R2-C2-M2-A2-N2-T2-E2-H2 (DS-1-like G2P[4]), G3-P[8]-I1-R1-C1-M1-A1-N1-T1-E1-H1 (Wa-like G3P[8]), and G9-P[8]-I1-R1-C1-M1-A1-N1-T1-E1-H1 (Wa-like G9P[8]). No atypical genotype constellation was detected except DS-1-like G1P[8] strains. Three mixed infection samples were detected: two seemed to be mixed infections of Wa-like G1P[8] and DS-1-like G1P[8], and one seemed to be a mixed infection of the Wa-like G1P[8] and G9P[8] strains.

During 2012–2014, DS-1-like G1P[8] (51.7%) was the most prevalent strain, followed by Wa-like G9P[8] (25.8%) (Table 1). Wa-like G1P[8], DS-1 like G2P[4], and Wa-like G3P[8] strains were detected in 12.6, 5.4, and 3.6% of cases, respectively. The reassortant DS-1-like G1P[8] strain is a newly emerged dominant genotype constellation, initially detected in 2012. This strain continued as to be dominant in 2013 and was detected in all six regions. In Aichi Prefecture, DS-1-like G1P[8] viruses were detected in 96.6% (86/89) of specimens. No DS-1-like G1P[8] was detected in 2014. However, only 14 RVA-positive specimens were detected in 2014. In particular, in Kyoto, no RVA-positive sample was detected in 2014 (0/15). On the whole, the genotype distributions varied drastically by region. For example, in 2013, DS-1-like G1P[8] was most prevalent in Sapporo, Akita and Aichi. Wa-like G1P[8] predominated in Kyoto and Yamaguchi, and G9P[8] predominated in Otaru (Table 1).

**Table 1:** Genotype distribution of RVAs in Japan (2012–2014).

Genotype constellation	Sapporo	Otaru	Akita	Aichi	Kyoto	Yamaguchi	Total
**2012 season^∗^**							
Wa-like G1P[8]	–	–	5	5	2	–	12 (11.1%)
DS-1-like G1P[8]	–	–	26	16	8	–	50 (46.3%)
DS-1-like G2P[4]	–	–	0	0	0	–	0 (0%)
Wa-like G3P[8]	–	–	2	3	3	–	8 (7.4%)
Wa-like G9P[8]	–	–	9	27	1	–	37 (34.3%)
Mixed	–	–	0	1	0	–	1 (0.9%)
Total	–	–	42	52	14	–	108 (100%)
**2013 season**							
Wa-like G1P[8]	1	0	0	1	12	15	29 (13.7%)
DS-1-like G1P[8]	14	4	6	86	8	4	122 (57.8%)
DS-1-like G2P[4]	5	4	2	0	0	0	11 (5.2%)
Wa-like G3P[8]	0	0	0	2	0	2	4 (1.9%)
Wa-like G9P[8]	6	28	0	0	1	8	43 (20.4%)
Mixed	0	0	0	0	1	1	2 (0.9%)
Total	26	36	8	89	22	30	211 (100%)
**2014 season (January – March)**							
Wa-like G1P[8]	–	–	0	1	0	0	1 (7.1%)
DS-1-like G1P[8]	–	–	0	0	0	0	0 (0%)
DS-1-like G2P[4]	–	–	0	3	0	4	7 (50.0%)
Wa-like G3P[8]	–	–	0	0	0	0	0 (0%)
Wa-like G9P[8]	–	–	2	2	0	2	6 (42.9%)
Mixed	–	–	0	0	0	0	0 (0%)
Total	–	–	2	6	0	6	14 (100%)
**Total of 2012–2014 seasons**							
Wa-like G1P[8]	1	0	5	7	14	15	42 (12.6%)
DS-1-like G1P[8]	14	4	32	102	16	4	172 (51.7%)
DS-1-like G2P[4]	5	4	2	3	0	4	18 (5.4%)
Wa-like G3P[8]	0	0	2	5	3	2	12 (3.6%)
Wa-like G9P[8]	6	28	11	29	2	10	86 (25.8%)
Mixed	0	0	0	1	1	1	3 (0.9%)
Total	26	36	52	147	36	36	333 (100%)


*^∗^Partially reported in Fujii et al. (2014).*

### Phylogenetic Analysis

Of 225 analyzed samples detected in 2013 and 2014, we completed nearly full-genome sequencing of 188 RNA samples (Supplementary Table S1). The remaining samples were excluded from phylogenetic analyses because the sequence data were incomplete (less than 80% length of at least one segment). With the 103 samples from 2012 (Fujii et al., 2014), 291 samples were used for phylogenetic analysis.

**FIGURE 1:**
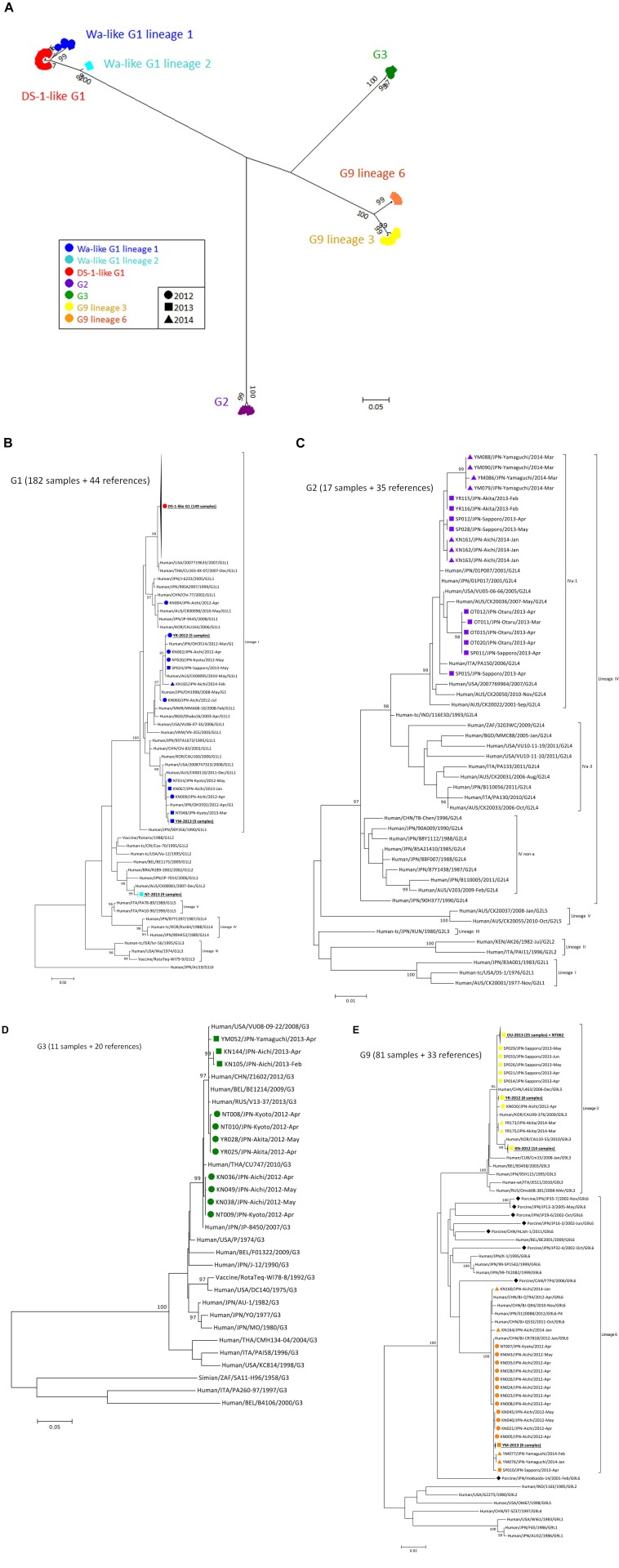
Phylogenetic tree of VP7 gene of RVA detected in Japan (2012–2014). A phylogenetic tree for VP7 was constructed from the nucleotide sequences of 291 RVA strains obtained in Japan in 2012–2014 and shown in radiation form **(A)**. All of Japanese strains were classified into seven major groups, and their markers were colored according to the groups: Wa-like G1P[8] lineage 1 (blue), Wa-like G1P[8] lineage 2 (light blue), DS-1-like G1P[8] (red), G2P[4] (purple), G3P[8] (green), G9 lineage 3 (yellow) and G9 lineage 6 (orange). The shapes of markers relate to the season of detection: 2012 (circle), 2013 (square) and 2014 (triangle). The tree of each VP7 genotype is shown with representative strains as references: G1 **(B)**, G2 **(C)**, G3 **(D)**, and G9 **(E)**. Similar strains are compressed to a triangle. Porcine-derived strains are marked by a diamond shape. The phylogenetic trees were constructed by using the Maximum Likelihood Method with 1000 bootstrap replicates. Percent bootstrap support is indicated by the value at each node when the value was 95% or larger.

To construct a VP7 tree (Figure 1A), all samples were classified into four genotypes: G1, G2, G3, and G9. Moreover, the G1 strains were divided into lineages 1 and 2, and G9 strains were divided into lineages 3 and 6. All of the DS-1-like G1P[8] strains were concentrated in one cluster of G1 lineage 1 (Figure 1A). When a detailed tree of each genotype was generated (Figure 1B–E), viruses of each genotype were further divided into multiple subclusters. Then, the subclusters were divided by region and season of collection. For example, Wa-like G1P[8] was divided into four subclusters (Figure 1B). One included nine strains from Kyoto in 2013 (NT-2013) and belongs to lineage 2. The other three subclusters belong to lineage 1; one consists of 149 DS-1-like G1P[8] strains, another is mainly composed of strains from Akita in 2012 (YR-2012), and the third is mainly composed of strains from Yamaguchi in 2013 (YM-2013). For the G2 tree (Figure 1C), SP011 and four strains from Otaru in 2013 were separated from other G2 strains, and four strains from Yamaguchi in 2014 were also different from other strains. For G3 tree (Figure 1D), NT009 and three strains from Aichi in 2012 were separated from other G3 strains. For lineage 3 of the G9 tree (Figure 1E), strains from Aichi in 2012 (KN-2012), Akita in 2012 (YR-2012) and Otaru in 2013 (OT-2013), including NT062, had similar sequences in each subcluster (nucleotide identities were > 99.8%).

**FIGURE 2:**
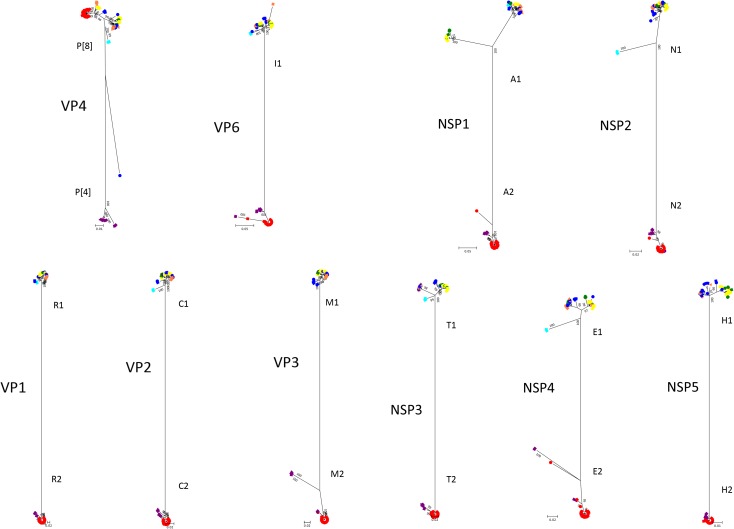
Phylogenetic trees of RVA genes other than VP7. Phylogenetic trees of 10 segments (VP4, VP6, VP1, VP2, VP3, NSP1, NSP2, NSP3, NSP4, and NSP5) were constructed from the nucleotide sequences of 291 RVA strains detected in this study, and shown in radiation form. The colors of markers indicate the groups of VP7 sequences, and the shapes of markers indicate the season in which they were detected (as in Figure 1). The phylogenetic trees were constructed by using the Maximum Likelihood Method with 1000 bootstrap replicates. Percent bootstrap support is indicated by the value at each node when the value was 95% or larger.

**FIGURE 3:**
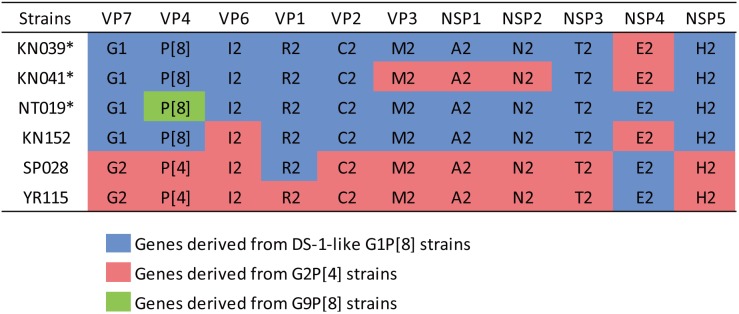
Further reassortant strains between DS-1-like G1P[8] and other strains. Six further reassortant strains were identified in 2012–2014, and their genotype constellations are shown. Asterisks indicate strains listed in our previous report (Fujii et al., 2014). Blue, red, or green color indicate the genes derived from DS-1-like G1P[8], G2P[4], or G9P[8] strains, respectively.

The phylogenetic trees of other gene segments are shown in Figure 2. Color dispersions show that intra-genogroup reassortments seemed to occur frequently, whereas inter-genogroup reassortment was not found except for the presence of DS-1-like G1P[8]. Reassortments among the Wa-genogroup strains (Wa-like G1P[8], Wa-like G3P[8], and Wa-like G9P[8]) were frequently found. Meanwhile, no reassortment was found in all segments of the G1 lineage 2 strains (light blue), which indicates a local epidemic in Kyoto in 2013. Some further reassortments were found between DS-1-like G1P[8] strains (red) and other strains. A total of six further reassortants including genes derived from DS-1-like G1P[8] strains were identified (Figure 3), and of these, three were already reported (Fujii et al., 2014). Of the rest, one was DS-1-like G1P[8] (KN152), and the two were G2P[4] strains (SP028 and YR115). KN152 reassorted with VP6 and NSP4 of G2P[4] strain, SP028 reassorted with VP1 and NSP4 of DS-1-like G1P[8] strain, and YR115 reassorted with NSP4 of DS-1-like G1P[8] strain. Reassortments seem to occur with greater frequency in NSP4 gene.

### Bayesian Analysis

**FIGURE 4:**
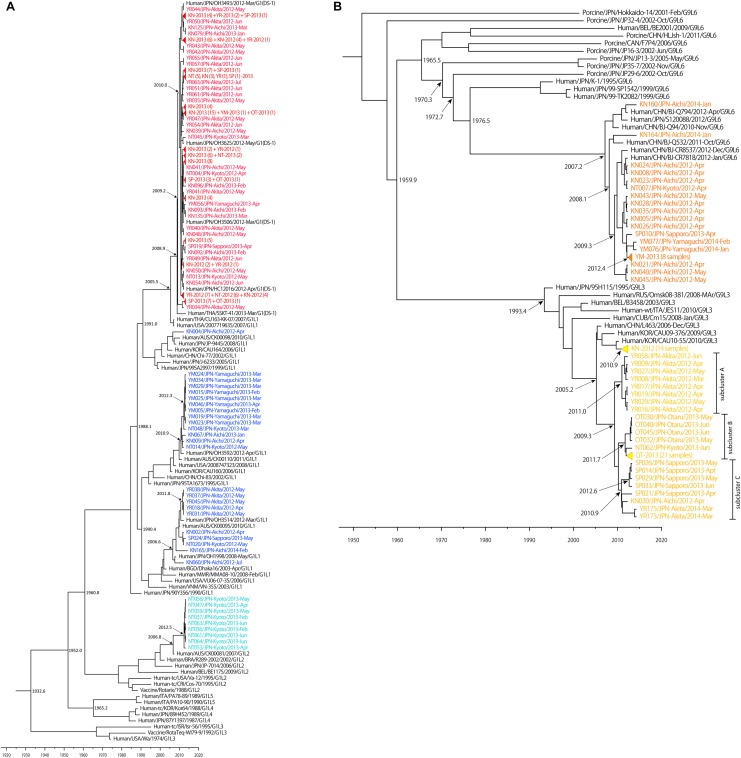
Maximum clade credibility (MCC) tree of VP7 constructed using the Bayesian MCMC method. Bayesian time-scaled trees of VP7 of G1 **(A)** and G9 **(B)** strains were generated, based on same data set as Figure 1. The estimated ages are indicated for particular nodes. Wa-like G1P[8] lineage 1 strains, Wa-like G1P[8] lineage 2 strains, DS-1-like G1P[8] strains, G9 lineage 3 strains and G9 lineage 6 strains detected in our study were colored: blue, light blue, red, yellow, and orange, respectively. Some similar strains were compressed to triangles.

A Bayesian time-scaled tree of VP7 was generated for each genotype with our 291 samples and the other 121 representative strains retrieved from GenBank (44 G1 references, 35 G2 references, 20 G3 references, and 26 G9 references) (Figure 4). The estimated evolution rates were 7.26 × 10^-4^ nucleotide substitutions per site per year (the 95% highest posterior density (HPD) was 5.99–8.63 × 10^-4^) for G1 strains, 7.41 × 10^-4^ (5.66–9.196 × 10^-4^) for G2 strains, 7.34 × 10^-4^ (4.69 × 10^-4^–1.01 × 10^-3^) for G3 strains, and 1.03 × 10^-3^ (7.46 × 10^-4^–1.38 × 10^-3^) for G9 strains.

The Bayesian time-scaled trees of G1 and G9 genotypes are presented in Figure 4. For the G2 and G3 trees, the sample numbers were too low to investigate. For the G1 tree (Figure 4A), all of the 149 DS-1-like G1P[8] strains were concentrated in one subcluster, and the DS-1-like G1P[8] strains detected in Okayama (Kuzuya et al., 2014), Osaka (Yamamoto et al., 2014), and Thailand (Komoto et al., 2015) were in same subcluster. The common ancestry of the DS-1-like G1P[8] strains was estimated to trace back to 2009.2 (2007.8–2010.5). This is 3 years (2–5 years considering 95% HPD) before this strain became a national epidemic in 2012. Meanwhile, the Wa-like G1 type was divided into three subclusters as described above (Figure 1B). For example, the time of most recent common ancestors (tMRCA) of the strains collected in Yamaguchi in 2013 was estimated 2012.3 (2011.5–2012.9). Thus, it was less than 1–2 years before the ancestor virus became epidemic in Yamaguchi. Two other subclusters (Akita in 2012 and Kyoto in 2013) showed similar patterns.

While the G9 genotype is divided into six lineages (Phan et al., 2007), only lineages 3 and 6 were circulating in Japan. Lineages 3 and 6 were estimated to have separated around 1960 (Figure 4B). Most of lineage 3 strains detected in Aichi in 2012 were clustered away from other strains detected in other places, and the tMRCA was estimated as 2010.9 (2010.0–2011.6). For descriptive purpose, the other lineage 3 strains were further divided into three subclusters (i.e., A, B, and C) (Figure 4B). For subcluster A, the tMRCA of eight strains from Akita in 2012 was 2011.0 (2009.6–2011.9). For subcluster B, the tMRCA of 26 strains from Otaru in 2013 except for NT062 was 2011.7 (2010.7–2012.5). Subcluster C consists of strains from Sapporo (2013), Akita (2014), and Aichi (2012), and the tMRCA was 2010.9 (2009.5–2012.0). In each case, tMRCAs were 1–3 years before the local epidemics. Their common ancestor was traced back to 2009.3 (2006.9–2010.9). This is 2–6 years before the national epidemic as well for DS-1-like G1P[8]. G9 lineage 6 strains have a common ancestry with human and porcine RVA until around 1970. After that, human G9 lineage 6 seemed to evolve independently. The now-circulating G9 lineage 6 strains in Japan were traced back to 2007.2 (2004.7–2009.0). Because these G9 lineage 6 strains were already detected nationwide before we started this study in 2012, it seemed to be less than 5 years (95% HPD: 3–8 years) before the national epidemic as well.

## Discussion

**FIGURE 5:**
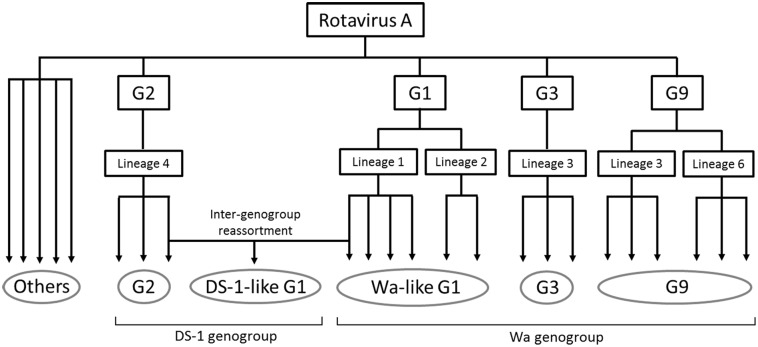
Simplified illustration indicating the RVA evolutionary history and epidemics in Japan in 2014. The five grouped RVAs based on genotype constellation are commonly circulating in Japan and are evolving by further dividing into multiple subclusters and sometimes by intra-genotype reassortment. DS-1-like G1P[8] strain exceptionally seemed to have generated by inter-genogroup reassortment between Wa-like G1P[8] strain and G2P[4] strain. “Others” means minor strains, such as G4, G8, G12, and so on.

In this study, NGS analysis revealed information about RVA epidemics in Japan from 2012 to 2014. Although there are limitations of collection period and non-homogeneous populations, 333 RVA-positive samples were collected from six sentinel hospitals across the country. All RVA strains were grouped into five patterns of genotype constellation except for three mixed infection cases (Figure 1). Using phylogenetic analysis, viruses were found to be further divided into multiple subclusters, and the strain distributions were different, depending on when and where they were collected (Figure 1B–E). This shows the obvious seasonal and geographical differences of RVA strain distribution at the sequence level. The evolutionary history and epidemics of RVA in Japan in 2014 are illustrated in Figure 5. Although more complex genotype distributions are found in developing and developed countries (Reidy et al., 2005; Chitambar et al., 2014; Dennis et al., 2014; Heylen et al., 2014; Nyaga et al., 2015), only five patterns of genotype constellation were detected in Japan. Without unusual strains, Japanese RVA epidemics can be explained with a simple model.

In 2012, DS-1-like G1P[8] was initially detected in Japan and seemed to be generated by reassortment of typical Wa-like G1P[8] and DS-1-like G2P[4] as previously described (Fujii et al., 2014). DS-1-like G1P[8] viruses were reported in Thailand (Komoto et al., 2015) and the Philippines (Yamamoto et al., 2017) in 2012, but it is difficult to determine where this strain came from. In Japan, DS-1-like G1P[8] was the predominant strain in 2012–2013, and six further reassortants between DS-1-like G1P[8] and others were detected (Figure 3). Of these, five occurred in the NSP4 segment. Thus, reassortment seems to occur more frequently in the NSP4 segment than other segments, and its evolutionary rate is higher than other segments (Tatsumi et al., 2014; Zeller et al., 2015). Therefore, the exceptional tendency for substitutions of NSP4 might affect the frequency of reassortments. DS-1-like G1P[8] was not detected in 2014, and G2P[4] accounted for half of RVA strains instead (Table 1). Several countries reported increased rates of G2P[4] after vaccine introduction (Leshem et al., 2014a; Wu et al., 2014; Montenegro et al., 2015). However, further investigation is required as the number of specimens in 2014 was small due partly to a shorter collection period and partly to an increasing use of RVA vaccine (Hashizume et al., 2015).

We found substantial differences in the distributions of genotypes, depending on where they were collected (Table 1). For example, in 2013, the predominant strains were DS-1-like G1P[8] in Sapporo and G9 lineage 3 in Otaru, even though these cities are only 30 km apart. Thus, RVAs might spread more slowly than noroviruses, another cause of gastroenteritis (Green, 2013). One reason may be that most RVA-associated gastroenteritis patients are infants. Infants are usually much less active than adults, and so, a viral strain has less chance to spread. Actually, the age distribution of norovirus infection cases is generally wider than that of RVAs, and noroviruses spread rapidly and show little difference in geographical distribution (Lindesmith et al., 2008; Siebenga et al., 2009; Green, 2013). Once an RVA strain is prevalent in a region, the population of infants there will acquire herd immunity in a few years, and the epidemic of that strain will subside. When the population without immunity to that strain (i.e., newborns) increases sufficiently, the strain could become prevalent again.

Next, a Bayesian time-scaled phylogenetic tree was constructed to understand the evolution of RVAs (Figure 4). The evolutionary rate was estimated to be 7.26 × 10^-4^ to 1.04 × 10^-3^ nucleotide substitutions per site per year for the G1, G2, G3, and G9 strains, which is similar to previous reports (Nagaoka et al., 2012; Afrad et al., 2014; Doan et al., 2015; Zeller et al., 2015). Analyses of tMRCA of prevalent RVA strains provided us a prediction of the spread speed of RVA strains. It takes 1–3 years from generation of parent viruses to be epidemic in a region and 1–3 years more to spread nationwide. Therefore, it takes 2–6 years from the generation of an ancestor virus for it to be an epidemic strain across Japan. Because DS-1-like G1P[8] strain emerged only in 2012, this method is appropriate for evaluating the evolutionary behavior of RVA. The tMRCA of DS-1-like G1P[8] strains was 2009.2 (2007.8–2010.5). Thus, it fits the pattern described above. Because RVAs were not comprehensively examined in Japan until 2012, the DS-1-like G1P[8] viruses might have been missed by the detection network. Although the DS-1-like G1P[8] virus was not detected again in 2014 in our study, this strain was circulating in Japan at least until 2015, according to another report (Sugimoto et al., 2017).

Here, we described the epidemics of RVA in Japan and elucidated the evolutionary behavior of RVAs. These findings will be important to predict epidemics of RVA. To monitor RVA epidemics reliably, a continuous nationwide surveillance is needed. As RVA gastroenteritis cases are decreasing owing to vaccine effect, the burden of analyses will become easier. Meanwhile, efficient sample collection will become more important in the future.

## Author Contributions

YF performed all experiments and was responsible for writing the manuscript. YD and YS gave assistance to perform phylogenetic analysis and Bayesian evolutionary analysis. TN and ON organized the sample collection network with hospitals. KK gave assistance for the research and helped to draft the manuscript.

## Conflict of Interest Statement

The authors declare that the research was conducted in the absence of any commercial or financial relationships that could be construed as a potential conflict of interest.

## Footnotes

1 http://tree.bio.ed.ac.uk/software/tracer/

2 http://tree.bio.ed.ac.uk/software/figtree/

## Supplementary Material

Figure S1 A map of Japan showing the sample collection sites and periods. The sites of hospitals joining in this study are indicated as black circles. The names of hospitals, prefectures, and collection periods (in parentheses) are shown.

Table S1 Sample list used for phylogenetic analysis.
