# Supplementary figures and images for: Study of Complete Genome Sequences of Rotavirus A Epidemics and Evolution in Japan in 2012–2014
Source: Front Microbiol. 2019 Jan 31;10:38. doi: 10.3389/fmicb.2019.00038 (PMC6365416; doi:10.3389/fmicb.2019.00038)

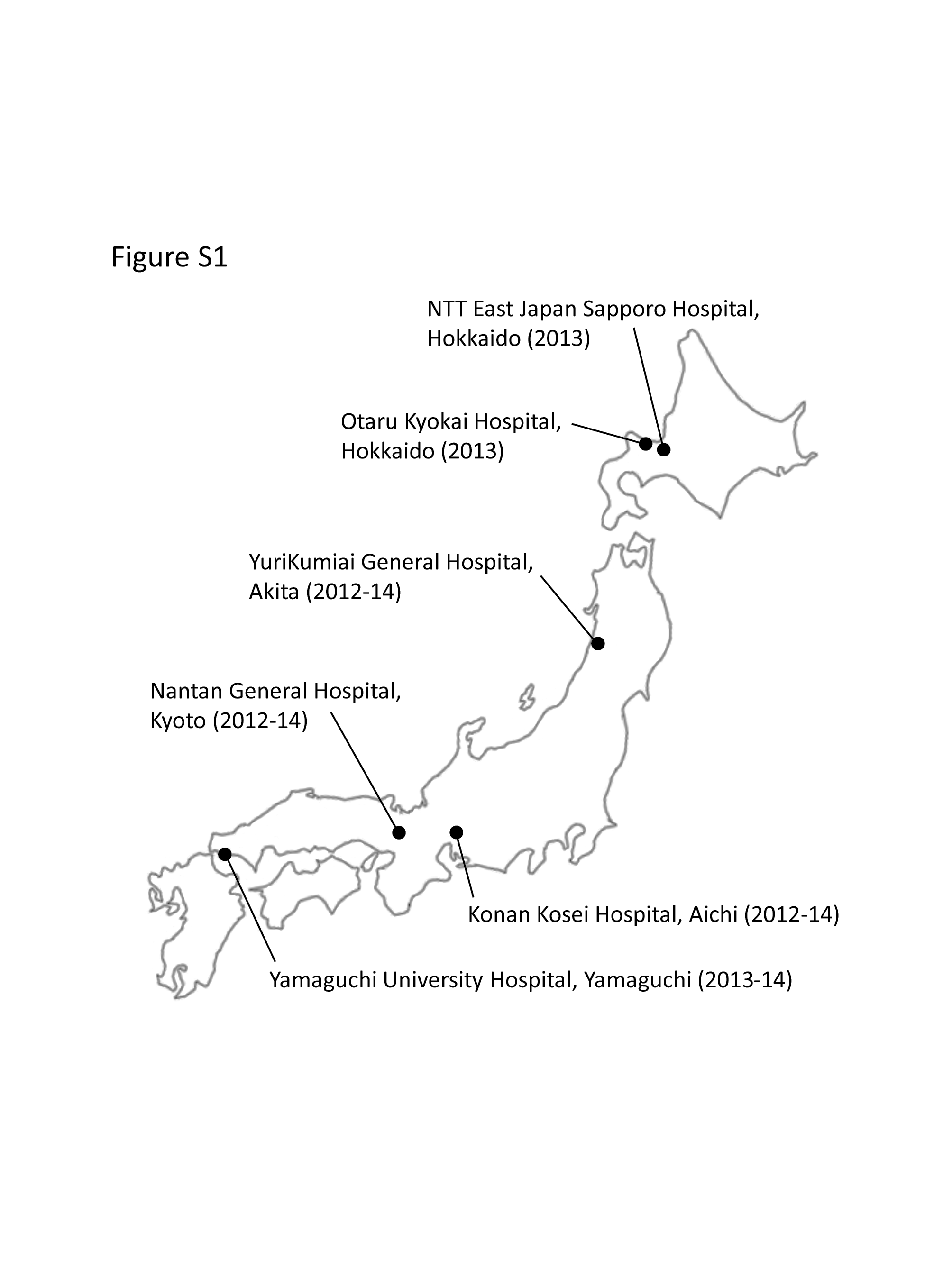

Supplement: Figure S1 — A map of Japan showing the sample collection sites and periods. The sites of hospitals joining in this study are indicated as black circles. The names of hospitals, prefectures, and collection periods (in parentheses) are shown. [file Image_1.TIF]
