# Supplementary material for: Study of Complete Genome Sequences of Rotavirus A Epidemics and Evolution in Japan in 2012–2014
Source: Front Microbiol. 2019 Jan 31;10:38. doi: 10.3389/fmicb.2019.00038 (PMC6365416; doi:10.3389/fmicb.2019.00038)
Supplement: Table S1 — Sample list used for phylogenetic analysis. [file Data_Sheet_1.PDF]

Table S1. Sample list used for phylogenetic analysis.

Genotype constellation	Sapporo	Otaru	Akita	Aichi	Kyoto	Yamaguchi	Total
2012 season							
Wa-like G1P[8]	-	-	5	4	2	-	11
DS-1-like G1P[8]	-	-	26	15	8	-	49
DS-1-like G2P[4]	-	-	0	0	0	-	0
Wa-like G3P[8]	-	-	2	3	3	-	8
Wa-like G9 L3	-	-	8	15	0	-	23
Wa-like G9 L6	-	-	0	11	1	-	12
Total	-	-	41	48	14	-	103
2013 season							
Wa-like G1P[8]	1	0	0	1	10	9	21
DS-1-like G1P[8]	14	3	3	70	8	2	100
DS-1-like G2P[4]	4	4	2	0	0	0	10
Wa-like G3P[8]	0	0	0	2	0	1	3
Wa-like G9 L3	5	25	0	1	0	0	31
Wa-like G9 L6	1	0	0	0	0	8	9
Total	25	32	5	74	18	20	174
2014 season (Jan. - Mar.)							
Wa-like G1P[8]	-	-	0	1	0	0	1
DS-1-like G1P[8]	-	-	0	0	0	0	0
DS-1-like G2P[4]	-	-	0	3	0	4	7
Wa-like G3P[8]	-	-	0	0	0	0	0
Wa-like G9 L3	-	-	2	0	0	0	2
Wa-like G9 L6	-	-	0	2	0	2	4
Total	-	-	2	6	0	6	14
Total of 2012-2014 seasons							
Wa-like G1P[8]	1	0	5	6	12	9	33
DS-1-like G1P[8]	14	3	29	85	16	2	149
DS-1-like G2P[4]	4	4	2	3	0	4	17
Wa-like G3P[8]	0	0	2	5	3	1	11
Wa-like G9 L3	5	25	10	16	0	0	56
Wa-like G9 L6	1	0	0	13	1	10	25
Total	25	32	48	128	32	26	291
